# Controlling my genome with my smartphone: first clinical experiences of the PROMISE system

**DOI:** 10.1007/s00392-021-01942-8

**Published:** 2021-10-25

**Authors:** Ali Amr, Marc Hinderer, Lena Griebel, Dominic Deuber, Christoph Egger, Farbod Sedaghat-Hamedani, Elham Kayvanpour, Daniel Huhn, Jan Haas, Karen Frese, Marc Schweig, Ninja Marnau, Annika Krämer, Claudia Durand, Florian Battke, Hans-Ulrich Prokosch, Michael Backes, Andreas Keller, Dominique Schröder, Hugo A. Katus, Norbert Frey, Benjamin Meder

**Affiliations:** 1 Institute for Cardiomyopathies, Department of Medicine III, University of Heidelberg, Im Neuenheimer Feld 410, 69120 Heidelberg, Germany; 2 DZHK (German Centre for Cardiovascular Research), 69120 Heidelberg, Germany; 3 Chair of Medical Informatics, Friedrich Alexander University Erlangen-Nürnberg, 91058 Erlangen, Germany; 4 Chair for Applied Cryptography, Friedrich-Alexander University Erlangen-Nürnberg, 90429 Erlangen, Germany; 5 Department of General Internal Medicine and Psychosomatic, University Hospital Heidelberg, 69120 Heidelberg, Germany; 6 Backes SRT GmbH, 66111 Saarbrücken, Germany; 7 CISPA Helmholtz Center for Information Security, 66123 Saarbrücken, Germany; 8 CeGaT GmbH, Center for Genomics and Transcriptomics, 72076 Tübingen, Germany; 9 Chair for Clinical Bioinformatics, Saarland University, 66123 Saarbrücken, Germany; 10 Chair for Information Security and Cryptography, Saarland University, 66123 Saarbrücken, Germany; 11 Stanford Genome Technology Center, Stanford University School of Medicine, Palo Alto, CA 94305 USA

**Keywords:** Digital health, Whole-genome sequencing, Genetic data transfer, Data security, Privacy, Big data democratization

## Abstract

**Background:**

The development of Precision Medicine strategies requires high-dimensional phenotypic and genomic data, both of which are highly privacy-sensitive data types. Conventional data management systems lack the capabilities to sufficiently handle the expected large quantities of such sensitive data in a secure manner. PROMISE is a genetic data management concept that implements a highly secure platform for data exchange while preserving patient interests, privacy, and autonomy.

**Methods:**

The concept of PROMISE to democratize genetic data was developed by an interdisciplinary team. It integrates a sophisticated cryptographic concept that allows only the patient to grant selective access to defined parts of his genetic information with single DNA base-pair resolution cryptography. The PROMISE system was developed for research purposes to evaluate the concept in a pilot study with nineteen cardiomyopathy patients undergoing genotyping, questionnaires, and longitudinal follow-up.

**Results:**

The safety of genetic data was very important to 79%, and patients generally regarded the data as highly sensitive. More than half the patients reported that their attitude towards the handling of genetic data has changed after using the PROMISE app for 4 months (median). The patients reported higher confidence in data security and willingness to share their data with commercial third parties, including pharmaceutical companies (increase from 5 to 32%).

**Conclusion:**

PROMISE democratizes genomic data by a transparent, secure, and patient-centric approach. This clinical pilot study evaluating a genetic data infrastructure is unique and shows that patient’s acceptance of data sharing can be increased by patient-centric decision-making.

**Graphic abstract:**

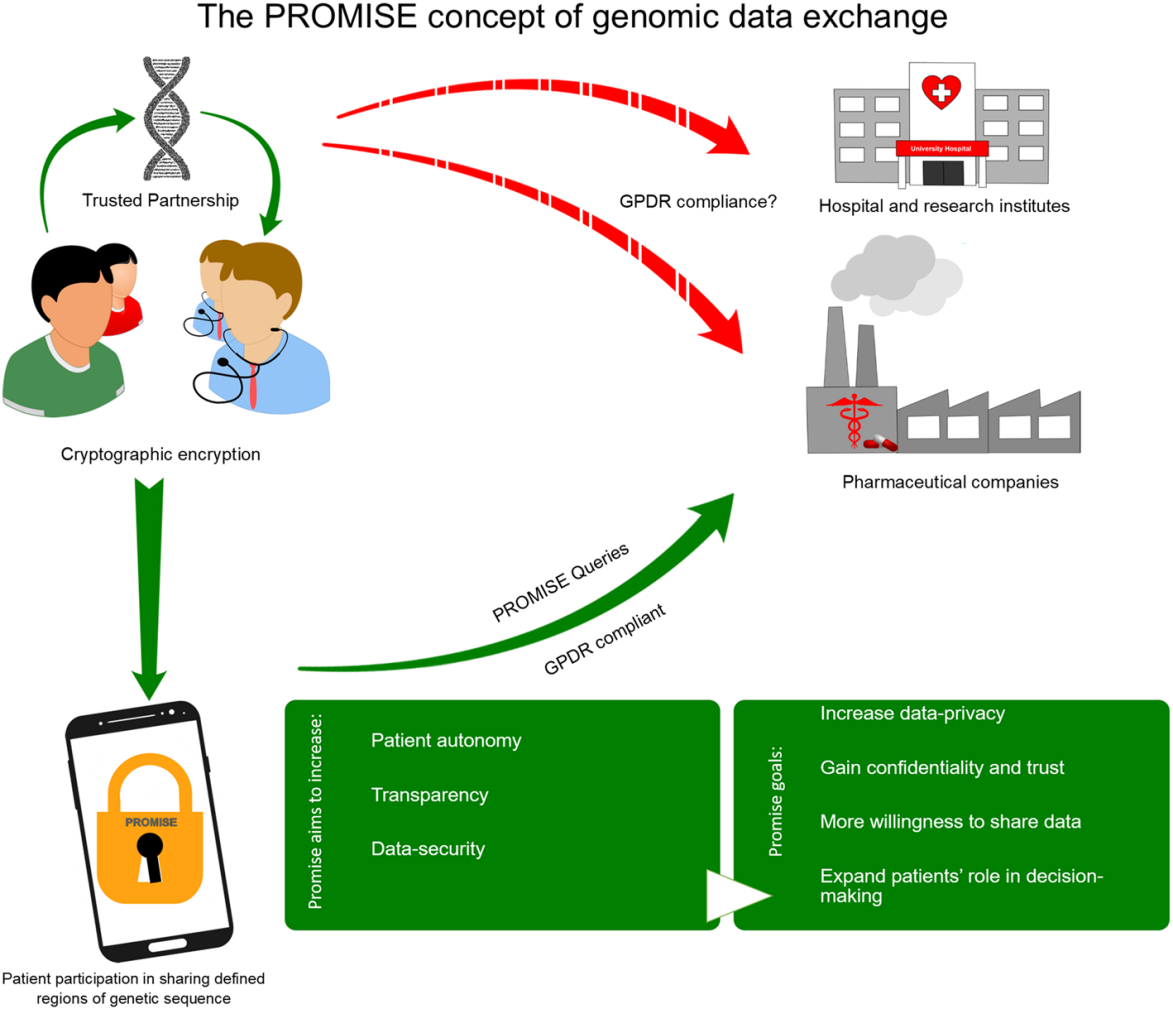

## Introduction

Genomics is a necessity for personalized medical care, and with the decrease in sequencing costs as well as the increased understanding of genetic factors, the integration of whole-genome sequencing (WGS) into the clinical routine and pharmaceutical research is imminent [1–4]. Genetic testing has uncovered the etiologies of many diseases that were previously classified as idiopathic [5, 6]. The transition from sequencing single genes to WGS has helped clinicians and researchers to gain a better understanding of disease pathomechanisms [7, 8]. Genomic data are regarded as extremely sensitive due to its unchangeable nature and the information it holds. Monogenetic diseases, susceptibility for multifactorial disorders, individual features like skin, hair, or eye color, and clues about the patient’s origin can currently be abstracted from genomic data [9–13]. The interpretation of data from WGS is, however, still complex and very often data is reanalyzed when additional knowledge becomes available [14–16]. Consequently, large quantities of genomic data have to be stored for longer periods of time.

Conventional data management systems lack the capabilities to adequately handle genome-scale quantities of highly sensitive data in a secure manner. Hence, although there might be a benefit for many patients having their genotyping information at hand, the risk taken regarding data security and privacy is relatively high [17]. The difficulties faced today are expected to even augment in the future [18, 19]. These issues demotivate patients to share their genomic data for important research purposes.

Medical care could be significantly improved through genetic data sharing between research centers and pharmaceutical companies [20]. Genomic data exchange is needed for the development and improvement of precise medicine strategies, with an exponential number of investigational drug trials requiring genotype information for patient selection [21–24]. Hence, the dilemma of genetic data sharing and its potential abuse must carefully and continuously be balanced [25–27]. As data security and privacy go hand in hand, the management of genomic data raises many questions concerning secure data storage, patient consent, and accessibility [28–31]. PROMISE is a novel secure genomic data management system, which offers a secure platform for genetic data exchange while preserving patient privacy and autonomy. The PROMISE-consortium consisted of the following project partners: University of Saarland, CISPA, Friedrich Alexander University Erlangen-Nürnberg, University Hospital Heidelberg, Backes:SRT GmbH, CeGaT GmbH and was comprised by an interdisciplinary team of clinical researchers, geneticists, legal scholars, computer scientists and engineers, medical and information technology researchers, and bioinformaticians. The project and consortium were supported and financed through the Federal Ministry of Education and Research (BMBF). The PROMISE application for Android OS was developed by the consortium for research purposes and is not yet available for end-users. The cryptographic protocol enables enhanced data privacy so that even the cloud server administrator cannot analyze the data without the explicit consent of the patient. Each DNA base-pair is encrypted by its own token, which enables the patient to provide third parties access to fine-grained regions of his genome without the risk of sharing unwanted information. The in-depth technical aspects of the cryptographic system are explained in detail in [32].

## Materials and methods

### Study design

The current pilot study aimed to investigate issues regarding the impact of data control by the patient, genetic sequencing, data storage, and analysis. Furthermore, the study assessed the patients' awareness and perception of data privacy and security and the effects on the patient-physician relationship. This is a single-center, non-randomized prospective study of the PROMISE application. Patients suffering from dilated, hypertrophic, or arrhythmogenic cardiomyopathy were enrolled in the study, and genetic testing by next-generation sequencing was performed after consenting. Patients in the study were introduced to the PROMISE app.

### Description of the PROMISE application

**Fig. 1:**
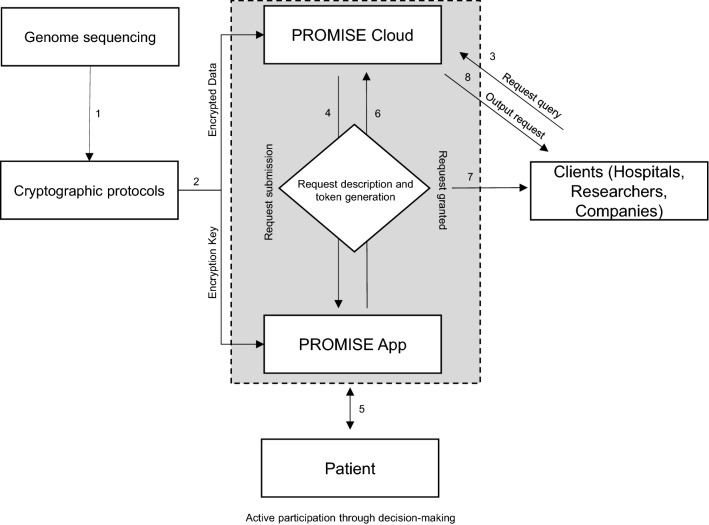
Overview of the PROMISE platform workflow. The genetic data are cryptographically encrypted after completion of the genetic sequencing (**1**). The encrypted data is transferred to the PROMISE Cloud for storage and the encryption key is transferred to the PROMISE app (**2**). This process is done only once and does not need to be repeated. Clients can send a data request to the PROMISE system (**3**). The request description with precise information on the requested data and the client are transferred to the PROMISE app (**4**). The patient has the option to accept or decline the request. A token that allows the decryption of the computation is generated for that specific query after the approval (**5**). The token is then encrypted and sent to the system and client (**6**). The client receives the encrypted key for that token (**7**). The computation of the data is performed on the still encrypted data. The results of the encrypted computation are sent to the client (**8**). The client can decrypt the output of the computation using the decrypted token received from the PROMISE app upon approval of the query. The token can only decrypt the computation for that very specific query and no other information of the patient is revealed, neither to the PROMISE Cloud nor the client

A high-level overview of the technical realization of the PROMISE system is represented in Fig. 1. The technical details of the underlying cryptographic system are published in [32] and is a variant of the well-studied protocol by Yao [33] for secure multi-party computation. The PROMISE system allows the interaction of different entities: the clinical sequencing center, patients, PROMISE cloud, and the “clients”. The clinical sequencing center is responsible for sequencing the genetic information and inserting it into the system. There can be many such sequencing centers, and no participation is required from them after inserting the data. The patient participates in the system as a “keyholder” authorizing individual computations using a mobile device like a smartphone. This immediately rules out any setting where the patients themselves need to store genetic information or perform computations. PROMISE itself acts as a cloud provider contributing storage and computational capabilities on behalf of the patient. The system design ensures that PROMISE never has access to the unencrypted genetic information. This immediately rules out any cloud system, which directly executes computation on unencrypted genetic data. Finally, the “client” is interested in a particular computation. Clients need to ask the patients for explicit consent for a particular computation. The client only gains access to the output of that specific computation request authorized by the patient. This rules out any system where the client is given direct access to (or parts of) the unencrypted genetic information.

The PROMISE system achieves the following desirable properties through this system design: (a) No single party is able to access the patient’s genetic information without that particular patient's active consent. (b) “Clients” do not gain access to any additional information about the patient's genetic information beyond that what was agreed to by the patient. (c) While the patient uses a mobile device to authorize computation requests, both the storage and the computation requirements on that device are minimal, and the loss of the device (alone) does not jeopardize the genetic information. (d) PROMISE facilitates the execution of the PROMISE system but does not, in itself, gain any information.

It is important to note that the properties of the PROMISE system are enforced cryptographically. This differentiates PROMISE's approach from cloud systems that focus on access control. In PROMISE, neither the cloud provider nor rouge system administrators can gain illegitimate access to the data. This is ensured since the genetic information is never decrypted, and only the exact information needed for a concrete and explicitly authorized request is accessible to computation. Concretely, only the result of the authorized computation and no intermediate information can be decrypted and only by the authorized client. We would like to stress that despite the strong cryptographic guarantees provided by the PROMISE system, this does not restrict the computations possible. If a patient consents to some computation on unencrypted information, then PROMISE can securely provide the same computation under realistic cost parameters.

An overview of the interaction of the PROMISE system is as follows: The genetic data are cryptographically encrypted after genetic sequencing. The encrypted data is transferred to the PROMISE Cloud for storage and the encryption key is transferred to the PROMISE app installed on the patient’s smartphone. A client who wishes to perform a computation on a patient's genetic information contacts the PROMISE system. The precise description of the query is subsequently sent to the patient's device with a request for authorization. The patient can then, potentially after seeking outside advice and consultation, consent to the computation and generate a token that allows the decryption of the computation. The token is then encrypted and sent to the client and PROMISE Cloud. The decryption key of the token is transferred to the client. The encrypted token allows the computation of the query on the encrypted genetic data. The client and PROMISE server can then compute the still encrypted result. The client can then decrypt the output of the computation using the decryption token provided by the patient. The token is useless for decrypting any computation that differs from the authorized one in any way. The privacy of the patient and his family members are preserved in this way. Familial genetic features are neither disclosed to the client or to PROMISE (data-holder). Furthermore, the patient has the option to remain anonymous to the "client" requesting the data. The access to the data is a single access only at that given time period.

### Ethics statement

The written study proposal and protocol have been formally submitted and accepted by the ethics committee of the Medical Faculty of the University of Heidelberg. The study proposal with the number S-260/2017 was accepted on 06.06.2017.

### Study protocol, inclusion, and exclusion criteria

**Fig. 2:**
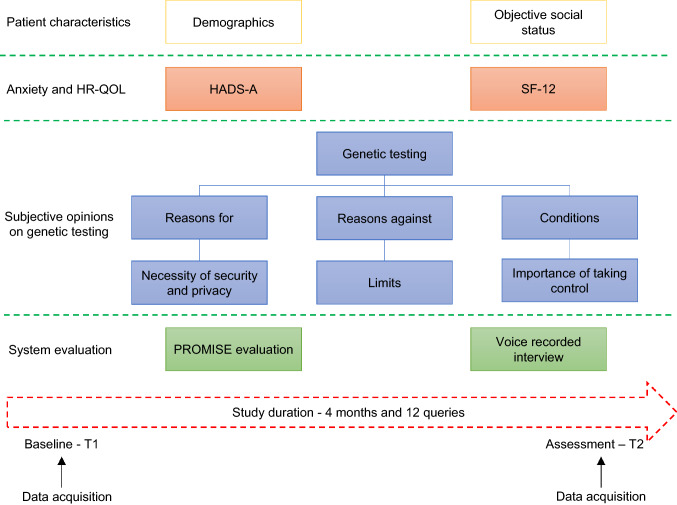
Schematic representation of the study protocol. The patient demographics are obtained from the clinical file at baseline (T1). Patients are requested to fill two validated questionnaires evaluating the anxiety status of the patient and the health-related quality of life: HADS-A and SF-12. The patients are also requested to complete a custom-made questionnaire and to take part in a voice-recorded questionnaire assessing their opinions on different topics regarding genetic testing and data sharing. After the training and installation of the mobile application, the patients receive up to 12 queries over a period of 4 months. A follow-up visit takes place after this time period (T2), where the patients are requested to take the same questionnaires at baseline again plus a questionnaire specifically evaluating PROMISE along with a voice-recorded interview

Patients > 18 years with a medical indication for genetic testing and were holders of a smartphone running on Android OS were consecutively enrolled in the study. Patients with clinically significant concurrent illness or psychological, familial, sociological, geographical, or other concomitant conditions that would not permit freely given informed consent were excluded. An overview of the workflow is shown in Fig. 2. The baseline patient demographics were obtained from the hospital's electronic clinical information system. The social status was assessed using a standard questionnaire. The Hospital Anxiety and Depression Scale (HADS-A) (7 items) and the Short Form 12 (SF-12) (12 items) are validated questionnaires which show a high internal consistency, good acceptability, and relatively low respondent burden [34–39]. These questionnaires were primarily used to detect relevant changes in the quality of life of patients to detect a possible influence on the patients' answers. The assessment of the patient's subjective opinion on genetic testing took place in the form of a custom-designed questionnaire and a voice-recorded interview. The questionnaire was created by the multidisciplinary expert team and tackles the following areas: the patient’s subjective knowledge of genetic testing, reasons for and against it, the necessary conditions needed for genetic testing, and the patient’s subjective opinion about the importance of taking control, data privacy and security. After completing the questionnaires, the patients were given a chance to expand on their ideas and subjective feelings through a voice-recorded session. They were also asked about their opinion on the utility of an electronic application as a mediator between the parties, the difficulties in using such a method of communication, causes for concern, and how it would affect the patient-physician relationship.

**Fig. 3:**
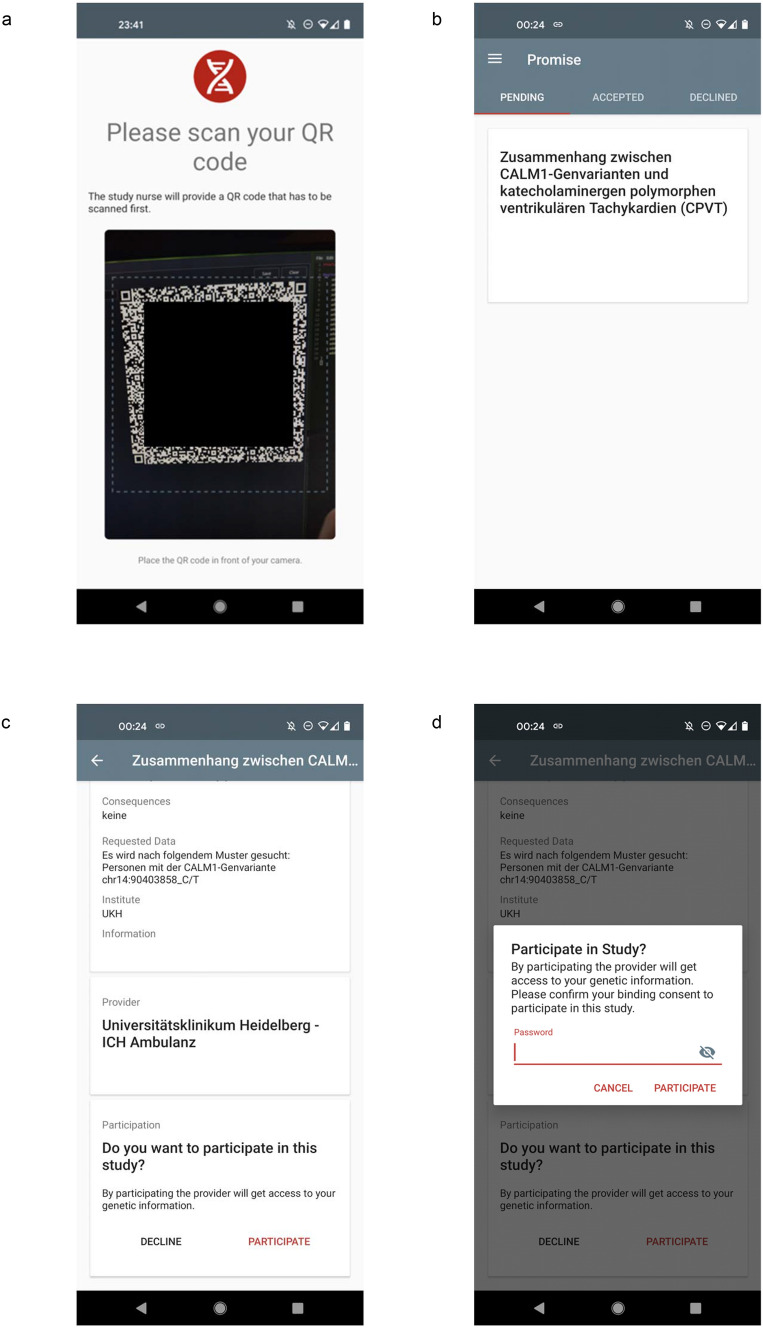
Exemplary screenshots of the PROMISE app. **a** A unique matrix code is generated at the initiation process at the sequencing center to register the smartphone to the patient. The encryption key to the data is sent to the patient once the smartphone is registered. **b**, **c** The patient can then receive requests from clients to access a specific part of his data. Detailed information regarding the request and the client can be displayed by clicking on the query. The patient has the opportunity to receive more information from the client and to request advice from his physicians. **d** A password is required to be entered to grant the request and to initiate the computation of the query

The installation, simulation, and application training were performed on completion of the baseline evaluation. Over the course of the study, which spanned over four months, each patient received up to 12 queries through the mobile application requesting parts of their genetic data (e.g., individual SNPs). The data requests were sent from “simulated” hospital researchers, university researchers, and commercial companies to assess the possibility of the request-origin affecting the patient’s decisions. The patient had three options; to accept, decline, or ignore the request. Exemplary screenshots of the PROMISE app and an in-app request are shown in Fig. 3. The decisions made by the patient regarding each query were documented.

The study participants were requested to revisit the study center for the concluding evaluation after four months of using the mobile application. They were requested to retake the questionnaires from the baseline evaluation and answer a new questionnaire specifically evaluating the experience with the PROMISE system. They also had the chance to elaborate on their experience in a voice-recorded session. Additionally, unplanned out-patient visits or telephone inquiries because of anxiety or insecurity feelings due to a request of the app were documented. In this way, the subjective opinions of the patients and their behavior is assessed in every step to identify possible problems, shortcomings, or benefits.

### Statistical analysis

Data from the standardized questionnaires were analyzed and interpreted as recommended by the respective authors. The patients underwent comprehensive clinical characterization, and a descriptive statistical analysis of the study population was performed. The comparison of categorical and nominal variables was performed using Fisher’s exact test or Chi-Square test. For observations that are temporal independent or dependent (time series/non-time series), parametric and non-parametric statistical methods were applied. The Wilcoxon–Mann–Whitney test was used to compare the differences between the patient answers before using the mobile application and thereafter. Multivariate Poisson regression methods were utilized for associations and comparisons of the response change within patient subgroups. A *p* value lower than 0.05 was considered as significant.

## Results

### Demographics of clinical cohorts for digital precision health trials

First, the demographic characteristics of patients visiting our study center over the past two years were analyzed to predict a typical patient population for such a concept. Dilated cardiomyopathy was the most common diagnosis with 72% (*n* = 968), followed by hypertrophic cardiomyopathy with 21% (*n* = 282), and non-compaction cardiomyopathy with 5% (*n* = 64). The mean age of the patient population was 53 years (*n* = 1347) (range 18–81 years), and the patients were predominantly male (67%). Guideline-directed genetic testing was performed in patients that did consent, which altogether leads to a total of 33% of all-comer cardiomyopathy cases being genotyped in our center. Since the PROMISE application was developed for Android OS only, we screened 210 consecutive patients regarding technical requirements. Approximately 62% of patients that did visit the out-patient clinic had a smartphone. 78% of these patients used an android based mobile operating system (mobile OS). An iOS-based mobile OS was reported by 18% of the patient, whereas only a minority of the patients used other mobile OS.

A total of *n* = 19 patients were consecutively enrolled in the prospective, single-center (University Hospital Heidelberg) pilot study. A summary of the patient’s clinical characteristics and disease-relevant genetic findings are presented in Table 1. An underlying genetic cause was found in 32% (*n* = 6) of the patients. Dilated cardiomyopathy was the most common clinical phenotype. The results of the quality-of-life questionnaires showed no significant differences at t1 and t2 (PCS-12 median 53.1 (t1) vs 52.9 (t2) *p* = 0.92, MCS-12 median 55.9 (t1) vs 52.3 (t2) *p* = 0.77, HADS-A median 5.0 (t1) vs 7.0 (t2) *p* = 0.107).

**Table 1:** Clinical characteristics of the patients and disease-relevant genetic findings

	Gender	Phenotype	Age	Age at disease onset	NYHA class	Ventricular arrhythmia	Atrial fibrillation	LV ejection fraction (%)	Gene	Variant
1	M	DCM	51	37	1	No	Yes	15	DSG	p.Arg49His
2	M	HCM	32	30	1	No	No	65	MYH7	p.Asp928Asn
3	M	HCM	34	25	2	No	No	65	–	–
4	M	DCM	53	37	1	No	No	35	MYH7	p.Arg1193His
5	M	DCM	58	45	2	No	No	49	–	–
6	F	HCM	35	31	2	No	No	60	–	–
7	M	DCM	35	20	2	No	Yes	52	EMD	p.Trp200Ter
8	F	DCM	34	26	2	No	No	44	–	–
9	F	DCM	32	18	1	No	No	40	–	–
10	M	HCM	23	14	1	No	No	59	MYBPC3	c.3490 + 1G > T
11	M	DCM	34	24	1	No	No	57	–	–
12	F	DCM	36	19	1	No	No	55	–	–
13	M	HCM	67	58	2	No	Yes	40	–	–
14	F	PPCM/DCM	50	45	2	No	No	54	–	–
15	F	DCM	57	52	1	No	No	50	–	–
16	M	ARVC	31	22	1	No	No	58	PKP2	c.2014-1G > C
17	F	DCM	32	25	2	No	No	48	–	–
18	M	DCM	27	24	1	Yes	No	58	–	–
19	F	DCM	43	38	2	Yes	Yes	50	–	–

**Fig. 4:**
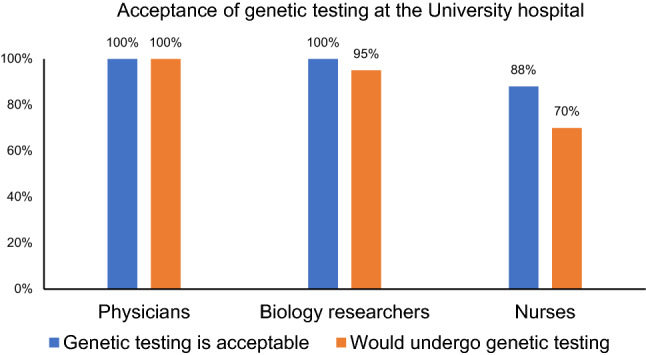
Graphical representation of the acceptance of genetic testing amongst the medical professionals at the university clinic. Medical professionals working at different capacities (physicians, biology researchers, and nurses) in different departments find that genetic testing is acceptable. The vast majority reported that they would undergo diagnostic genetic testing if recommended by the treating physician

The baseline questionnaire shows that most patients find genetic testing reasonable and useful (79%, *n* = 15). Furthermore, the majority of patients judge that genetic testing and research could have a positive impact on healthcare and biomedical research (89%, *n* = 17). Most of the patients regarded genetic data as very sensitive data, and 79% (*n* = 15) stated that the safety of genetic data is a very important issue. They reasoned that genetic data should be stored in a highly secure environment. In addition, at least 74% (*n* = 14) of patients judged that the PROMISE security concept protects their privacy even before actually using the system. Medical professionals working in different roles (physicians, medical biology researchers, and nurses; *n* = 24) at the university center were asked the same questionnaires. The vast majority of physicians and medical biology researchers were in favor of broad genetic testing and were willing to undergo diagnostic genetic testing if recommended by a physician Fig. 4.

### The PROMISE study shows the changing perception of patients after actively taking part in decision-making

**Fig. 5:**
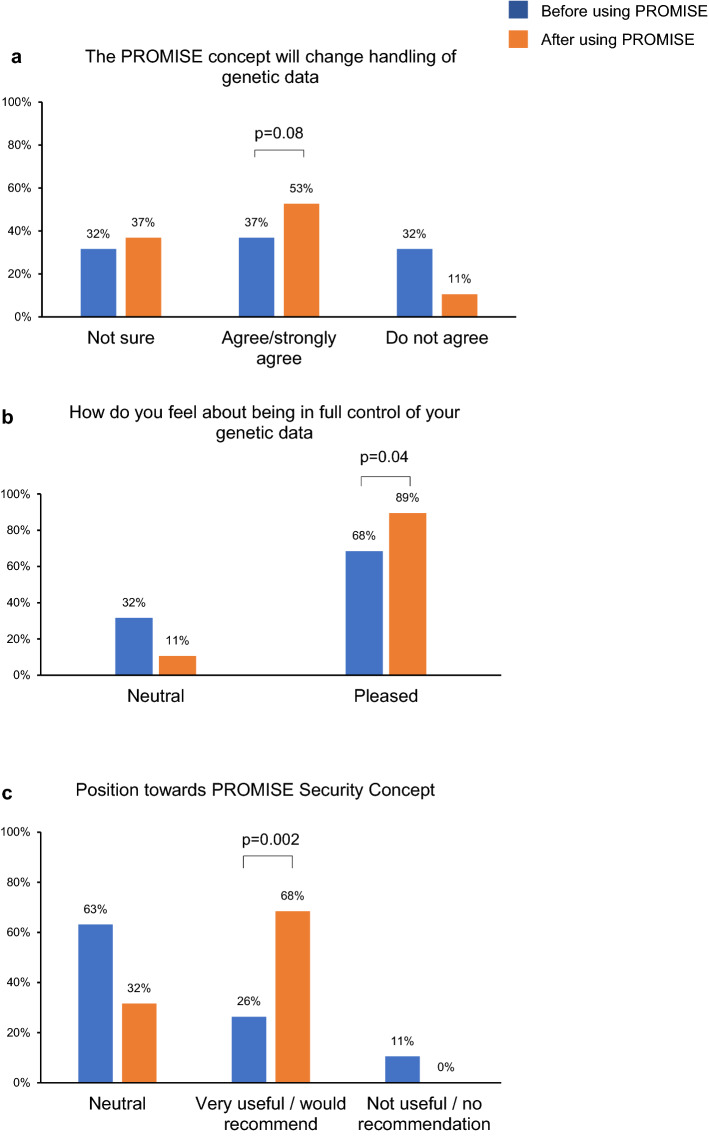
Graphical representations of the patients' opinions before and after using the PROMISE system. The concept of PROMISE was introduced to the patients at baseline. The blue bars represent the data acquired before using the PROMISE app (T1), while the orange bars represent the data acquired after using the PROMISE app (T2). The patients were asked if the concept of PROMISE would change their attitude towards the handling of genetic data (**a**), to give their opinion regarding being in full control of the genetic data (**b**), and their position towards the security concept of PROMISE (**c**)

The attitude and results of structured questionnaires were compared between the beginning (t1) and after using the cryptographic system (t2). Already after getting introduced to the PROMISE concept, 37% (*n* = 7) of patients already reported that their perception of genetic data storage and handling has changed due to the provided information by a specialist. The number increased to 53% (*n* = 10) after actually using the PROMISE app (Fig. 5a). Taking control of the genetic data has been perceived positively by the majority of the patients, especially after using the PROMISE app by themselves (68% (*n* = 13) before using the PROMISE app, 89% (*n* = 17) after participation in the clinical study) (Fig. 5b). The majority of the patients (63%; *n* = 13) were neutral towards recommending and using the system on a daily basis prior to using the PROMISE app. The number of individuals recommending such a system markedly increased from 26% (*n* = 5; t1) to 68% (*n* = 13; t2) during the follow-up (Fig. 5c).

The perceived active role of patients was reflected in 47% (*n* = 9) who were not at all concerned by taking full control of their own data. 74% (*n* = 14) of the patients were interested in receiving the results of the queries that were made by simulated clients during the study. However, the majority (63% *n* = 12) was also somehow concerned when receiving such notifications that included findings and interpretations of their genetic sequence, e.g., the susceptibility to distinct diseases targeted in an investigational drug study. Several participants pointed out in the interviews that it is very important for them to have a personal contact who supports them in using the app and handling requests for their data.

**Fig. 6:**
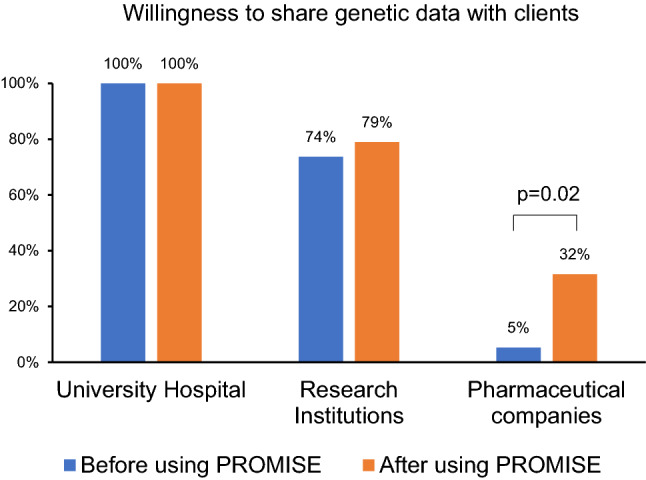
Figures representing the patient’s perspectives of different aspects after using the PROMISE system. The patients were questioned about their willingness to share their genetic data with different entities (university hospitals, research institutions and pharmaceutical companies). The blue and orange bars represent the responses of the patients before (T1) and after (T2) using the PROMISE app

To evaluate the most sensitive aspect of the PROMISE system, patients were asked about their willingness to share their genetic data. Most of the patients were willing to share their genetic data with university hospitals and research institutions (comparable at t1 and t2) (Fig. 6). However, only a very small minority of the patients (5%; *n* = 1) were willing to give pharmaceutical companies access to their genetic data before using the app. Interestingly, the number significantly increased to 32% (*n* = 6) after the study, indicating that trust in the platform and active decision-making is an important motivation for data sharing.

## Discussion

Translational biomedical research profoundly relies on the generation, exchange, and analysis of genomic data. Probands donating their highly sensitive genetic information ask for novel, privacy-sensitive approaches to avoid risks of data theft and misuse [40–43]. In the current clinical study, we evaluated a novel concept to store genomic data securely and democratize the utilization of data via an app-based system.

Genetic data hold information about the biological make-up of a given patient, his genetic diseases, medical and non-medical risk factors. From an ethical perspective, patients sharing their genetic data with research communities carry a disproportionate risk for their privacy [17, 44]. Conventional methods of genetic data storage and management either struggle to handle large quantities of data or rely on security concepts based on trust in institutions or companies [18, 45, 46]. In the real world, malicious activities, however, cannot be ruled out, and databases with genetic data of hundreds or thousands of individuals will be a hot target for malicious adversaries. The lack of physical control over data and, to a certain degree, missing transparency regarding data utilization could understandably restrict patients' willingness to share their genomic data with research communities or private entities [47, 48]. Explorative research showed that patients appear to generally support genomic data sharing but showed concern regarding data privacy and unauthorized utilization [49]. Previous research has also highlighted the importance of genetic data control by the patients themselves. Having such autonomy over the data was perceived as a form of safeguard against data misuse. Especially young adults were particularly interested in decisional privacy [50, 51]. The publicly funded project PROMISE developed a solution for these problems by providing a system of data transparency, security, and privacy, thereby reducing the risks carried by the patient while providing a high level of autonomy to decide on the where and when of data usage.

Recently, several user-centric genomic data sharing and analytics platforms have been proposed. Some of those platforms use blockchain cryptography to guarantee data integrity via distributed immutable ledgers and user control via smart contracts [52, 53]. Our approach does not utilize blockchain technology since they pose inherent privacy risks and conflict with the core requirements of the GDPR [54, 55]. PROMISE tries to solve fundamental issues by data democratization. Contrary to conventional data storage/management solutions (i.e., databases, cloud-servers, HER), PROMISE distinguishes itself especially in two main features: First, the data-holder (PROMISE) has no access to the genomic data (even if assumed corrupted with malicious intentions). Secondly, even though the data-holder (PROMISE) cannot access or decrypt the data, complex computations and queries can be performed on the data to disclose the results of the authorized query. Only the cryptographic interaction between the different elements in PROMISE enables the secure and private exchange of data between the patient and other parties (universities, research facilities, pharmaceutical companies, etc.). This method increases the autonomy of the patient and offers more transparency regarding data processing and analysis.

Currently available solutions for genetic data sharing are mainly driven by companies, and the consequences of introducing such systems are under-researched. The significant increase in autonomy is, in theory, advantageous for the patient, yet the shift of responsibility might also have negative consequences. Moreover, the repeated exposition to medical/research might lead to anxiety and decision fatigue. On the other hand, the patients' feelings of security and confidence in the health care system could be boosted through having tight control over their data. Furthermore, the ability to help the research community and public health through actively taking part in research projects could motivate patients to use the platform and have an additional positive effect on the patient’s psychological well-being. The majority of the patients in this study were aware of the potential benefit of genetic testing in clinical diagnostics and health care. At the same time, they were surprisingly aware of the sensitive nature of genetic data, and the majority agreed that it should be stored in a highly secure fashion. The concern of sharing their genetic data was reiterated in the patient interviews by the majority of participants. The patient’s experience of the PROMISE mobile application as a health app is published in [56].

Before using the PROMISE system, 68% of the patients reported positively about having full control of the data. A significant increase could be seen after the patients used the system. Similarly, a significant increase in the number of patients seeing a practical use for the system could be documented. Patients accentuated the importance of being able to decide, which resulted in an increased willingness to share their genetic data even with third parties such as companies. This is an important result of this study and should further motivate the active inclusion of the patient in data sharing concepts.

The current study highlights the awareness of patients regarding the sensitivity of genetic data and the importance of data safety from their perspective. The change in the attitudes of patients towards the management of genetic data is perhaps best reflected by the willingness to share their “sensitive” data with research entities. The number of patients consenting to data exchange with pharmaceutical companies increased significantly after using the PROMISE system. At the same time, the patients reported an increased sense of privacy and data safety. Being an active decision-maker in the process of data management was seen positively by the majority of the patients. The increased autonomy appears to increase the patient’s motivation in sharing their genetic data with research entities, which resonates with previous research results [50].

Potential limitations include the limited number of enrolled participants in this pilot study of the PROMISE system. It must be emphasized that each aspect was detailed in the study protocol, including genetic counseling, sequencing, training of the app, and PROMISE concept and data queries that simulated different client entities. In the future, a study with a larger number of participants is needed to validate the results. Another limitation is a possible selection bias since the patients who give their consent to take part in a research project that includes cloud-data processing of their genetic information are possibly less reserved regarding genetic testing and data sharing.

## Conclusion

The current study substantiates the value of self-participation concepts in eHealth applications. Genomic data are those with the highest demands for secure handling and advanced privacy concepts. PROMISE underlines that sophisticated technical solutions and cryptographic concepts have matured enough to enter the clinical arena and can provide an advantage for patients, doctors, and third parties for research and development.

## Data Availability

Anonymized data from the pilot study are available upon request.

## Code availability

The smartphone app and code are not freely available. Details of the cryptographic approach and code can be made available upon request.
